# Identification and molecular characterization of a novel flavin-free NADPH preferred azoreductase encoded by *azoB *in *Pigmentiphaga kullae *K24

**DOI:** 10.1186/1471-2091-11-13

**Published:** 2010-03-16

**Authors:** Huizhong Chen, Jinhui Feng, Ohgew Kweon, Haiyan Xu, Carl E Cerniglia

**Affiliations:** 1 Division of Microbiology, National Center for Toxicological Research, US Food and Drug Administration, Jefferson, AR 72079-9502, USA

## Abstract

**Background:**

Microbial degradation of azo dyes is commonly initiated by the reduction of the azo bond(s) by a group of NADH or NADPH dependant azoreductases with many requiring flavin as a cofactor. In this study, we report the identification of a novel flavin-free NADPH preferred azoreductase encoded by *azoB *in *Pigmentiphaga kullae *K24.

**Results:**

The deduced amino acid sequence of *azoB *from *P. kullae *K24 showed 61% identity to a previously studied azoreductase (AzoA) from the same strain. *azoB *encoded a protein of 203 amino acids and heterologously expressed in *Escherichia coli*. The purified recombinant enzyme was a monomer with a molecular mass of 22 kDa. Both NADH and NADPH can be used as an electron donor for its activity with 4-(4-hydroxy-1-naphthylazo) benzenesulfonic acid (Orange I) as substrate. The apparent *K*_m _values for both NADH and Orange I were 170 and 8.6 μM, respectively. The *K*_m _of NADPH for the enzyme is 1.0 μM. When NADPH served as the electron donor, the activity of the enzyme is 63% higher than that when NADH was used. The pH and temperature optima for activity of the enzyme with Orange I as the substrate were at pH 6.0 and between 37 and 45°C. Phylogenetic analysis shows that AzoB belongs to the flavin-free azoreductase group which has a key fingerprint motif GXXGXXG for NAD(P)H binding at the N-terminus of the amino acid sequences. The 3D structure of AzoB was generated by comparative modeling approach. The structural combination of three conserved glycine residues (G_7_xxG_10_xxG_13_) in the pyrophosphate-binding loop with the Arg-32 explains the preference for NADPH of AzoB.

**Conclusion:**

The biochemical and structural properties of AzoB from *P. kullae *K24 revealed its preference for NADPH over NADH and it is a member of the monomeric flavin-free azoreductase group. Our studies show the substrate specificity of AzoB based on structure and cofactor requirement and the phylogenetic relationship among azoreductase groups.

## Background

Azo dyes are characterized by one or more azo bonds (R-N = N-R) that allow visible light to be absorbed by the dyes. These dyes are used in a wide variety of consumer products including textile, paper, cosmetics, pharmaceuticals, and food [1]. Azo dyes such as Sudan dyes are not legal for use as colorants in foods, however, they recently have been detected as contaminants in the food supply [2]. The human health impact of exposure to azo dyes used in certain food products has caused concern since they may have genotoxic properties. The environmental fate and subsequent heath effects of the azo dyes released in textile and paper industry wastewater are increasing being studied by the scientific community [3].

While azo dyes are generally considered to be persistent pollutants because they are typically recalcitrant to aerobic biotransformation [4,5], they may be metabolized by azoreductases from commensal microorganisms, mammalian liver cells, and soil microorganisms [6]. A variety of microorganisms, including bacteria and fungi, are capable of decolorizing a diverse range of azo dyes. Some bacteria have the ability to degrade azo dyes both aerobically and anaerobically [2,6]. Bacterial degradation of azo dyes is often initiated by cleavage of azo bonds by azoreductases which are followed by the aerobic degradation of the resulting amines [5]. Two types of oxygen-insensitive azoreductases have been identified in bacteria: one is monomeric flavin-free enzymes containing a putative NAD(P)H binding motif and the other is polymeric flavin-dependent enzymes [5]. The genes encoding oxygen-insensitive flavin dependent azoreductases have been cloned from *Bacillus sp*. OY1-2 [7], *Escherichia coli *[8], *Enterococcus faecalis *[9], *Rhodobacter sphaeroides *[10], *Pseudomonas aeruginosa *[11], and *Staphylococcus aureus *[12]. Biochemical characteristics of bacterial FMN-dependent azoreductases and the protein structures of several enzymes have been recently determined [11-14]. Moreover, the role of specific amino acid residues involved in flavin binding and catalytic mechanism of oxygen-insensitive flavin dependent azoreductase from *E. faecalis *has been analyzed [15].

Two monomeric flavin-free azoreductases from *Xenophilus azovorans *KF46F [16] and *Pigmentiphaga kullae *K24 [17,18] have been described. However, little is known about the structure and function of monomeric flavin-free azoreductases from bacteria. *P. kullae *K24 was first described to contain oxygen-insensitive flavin-free azoreductase. This soil bacterium was isolated by long-term adaptation in the chemostat for growth on Orange I as the sole source of carbon and energy [18]. As a part of structure and function study of monomeric flavin-free azoreductases, we describe in this study the cloning and identification of a gene, *azoB*, which encodes a novel flavin-free NADH/NADPH dependent oxygen-insensitive azoreductase from *P. kullae *K24.

## Results

### Cloning of an azoreductase gene (*azoB*) from *P. kullae *K24

**Figure 1:**
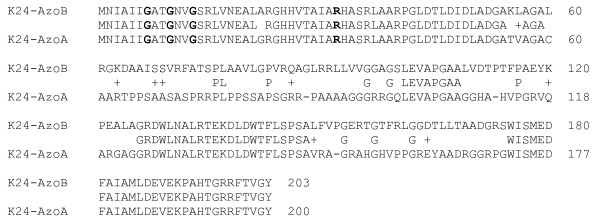
**Comparison of the deduced amino acid sequences of *P. kullae *K24 AzoB (top) and AzoA (bottom)**. Amino acid residues with an identical match (letter) and those with different degrees of conservation (+) are indicated. The NADPH binding residues are boldfaced.

An azoreductase gene, designed as *azoB*, was amplified from genomic DNA of *P. kullae *K24 by PCR using a pair of primers (K24NEW-forward and -reverse) yielding a DNA band of about 800 bp (812 bp) on agarose gel (not shown). It was directly ligated to TA cloning vector, pCR2.1-TOPO. Sequencing of the insert revealed that it contained 812 bp DNA fragment with a complete ORF (*azoB*). *azoB *encoded a protein consisting of 203 amino acid residues of 21,295 Dalton. The deduced protein sequence of AzoB displayed 61% primary structure identity to *P. kullae *K24 azoreductase A (AzoA) and had three amino acid residues more in size than that of the AzoA (AzoA, 200 amino acid residues, AY165002). Three deletion and/or insertion differences between these two protein sequences were found (Figure 1).

### Functional expression of the azoreductase in *E. coli*

*azoB *was inserted via its unique *Nde*I and *Bam*HI restriction sites into corresponding sites of pET-11a. The enzyme (AzoB) was functionally expressed as a native enzyme without modification in *E. coli *with a phage T7-promotor system. Crude-cell extracts were prepared from cultures of *E. coli *BL21-Gold(DE3)pLysS carrying the expression plasmid pAZOB, which had been induced by the addition of 0.1 mM IPTG for 2.5 h. Supernatant of the cell-extract from the induced culture displayed an elevated level of azoreductase activity (2.9 U/mg) in comparison with that of the non-induced culture (undetectable), indicating that the enzyme was induced and functionally expressed in the host.

### Purification of AzoB of *P. kullae *K24 from the recombinant *E. coli*

**Figure 2:**
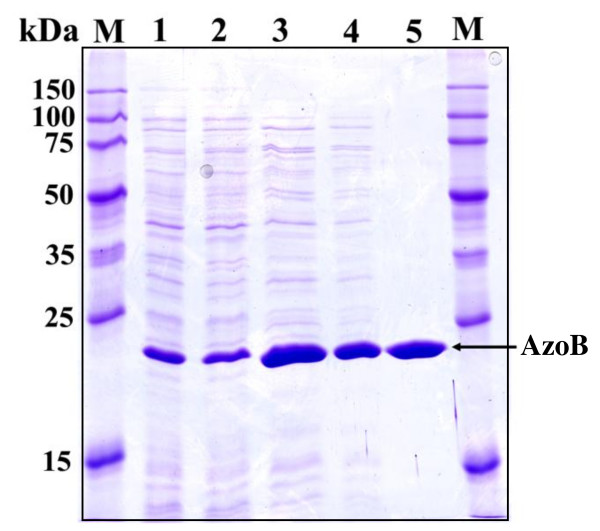
**Purification of *P. kullae *K24 AzoB from the recombinant *E. coli***. Lane M, protein size markers; lane 1, extraction of the recombinant *E. coli*; lane 2, ammonium sulfate deposition suspension; lane 3, pooled fractions with phenyl Sepharose FF; lane 4, fraction of concentrated sample of the pooled fractions with phenyl Sepharose FF; lane 5, pooled fractions with HiPrep SP XL.

The azoreductase was purified from the supernatant of the IPTG-induced recombinant *E. coli *by a combination of hydrophobic interaction chromatographies and ion exchange columns (Table 1). The enzyme was purified nearly 3.5-fold with a yield of 45%, which allows the proportion of the enzyme to be estimated at 28.7% of the total protein present in the crude cell extract. The specific activity of the purified *P. kullae *K24 AzoB was 10.1 U/mg protein using Orange I as the substrate. SDS-PAGE analysis showed that the molecular weight of the purified azoreductase was 22 kDa (Figure 2). Gel filtration chromatography on a HiLoad Superdex 75 column conformed the molecular weight obtained by SDS-PAGE. In comparison to that of AzoA (2.8 U/mg) from *P. kullae *K24 the enzyme has a high specific activity (10.1 U/mg). Only Orange I can be reduced by AzoB, while Methyl Red, Amaranth, Ponceau BS, Ponceau S, Orange II, Orange G, Megneson II, 1-(4-Nitrophenylazo)-2-naphthol, and 4-(4-Nitrophenylazo)-resorcinol were not reduced. The purified AzoB is colorless and no absorption spectrum of flavins was detected in the enzyme solution.

**Table 1:** Summary of purification of azoreductase from the recombinant *E. coli*^a^

Step	Total protein (mg)	Total activity (U)*	Specific activity (U/mg)	Yield (%)
Crude cell-extract	258	750.5	2.9	100%
Phenyl Sepharose FF	57.6	570.0	9.9	76%
HiPrep SP XL	33.2	334.3	10.1	45%

^a^Activities were determided with NADH as proton donor.

### Properties of the recombinant AzoB of *P. kullae *K24

**Figure 3:**
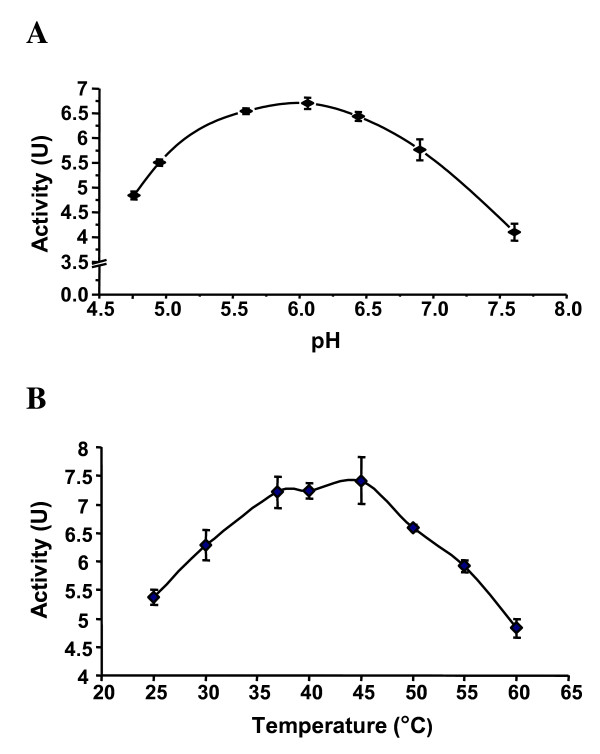
**Optimum pH and temperature of *P. kullae *K24 AzoB from the recombinant *E. coli***. A, the optimum pH. B, the optimum temperature. Data were from triplicate assays.

Enzymatic reactions were carried out by varying the concentration of one substrate and fixing the other substrate concentration at the same time. Analysis of the purified AzoB indicated the enzyme used both NADH and NADPH for Orange I reduction. Apparent *K*_m _and *V*_max _values were obtained from Lineweaver-Burk plots. The *K*_m _values for NADH and Orange I are 170 and 8.6 μM, respectively (Table 2). When NADPH served as the electron donor, the activity of the enzyme is 63% higher than NADH. The *K*_m _of NADPH for the enzyme is about 1.0 μM. When the enzyme activities were carried out in 50 mM Sorensen's phosphate buffer with different pH values and Orange I as substrate, the optimum pH was found to be around 6.0, as shown in Figure 3A. Enzyme activities were measured under different temperature from 25°C to 60°C. As shown in Figure 3B, the optimum temperature of the enzyme was found to be 45°C.

**Table 2:** Michaelis constants (*K*_m_) and maximal velocities (*V*_max_) of recombinant AzoB of *P. kullae *K24 expressed in *E. coli*.

Substrate	*K*_m _(μM)*	*V*_max _(U/mg protein)
NADPH	1.0	33
Orange I (NADPH)	3.0	36
NADH	170	17
Orange I (NADH)	8.6	22

* Data were presented by the averages from triplicate with standard deviations of <10%;

### *In silico *analysis of AzoB from *P. kullae *K24

**Figure 4:**
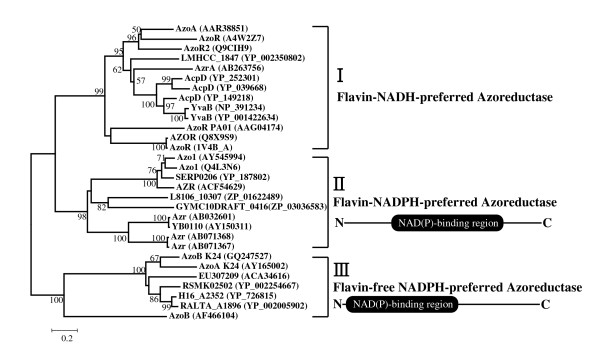
**Grouping of 30 azoreductases or hypothetic azoreductases with conserved dinucleotide binding domain**. The phylogenetic tree, inferred using the Neighbor-Joining method, shows the relative position of AzoB among other azoreductases. Bootstrap values for 100 replicates are given. Protein sources and GenBank accession numbers are as follows. AzoA (AAR38851)_*Enterococcus faecalis*; AzoR (A4W2Z7)_*Streptococcus suis *98HAH33; AzoR2 (Q9CIH9)_*Lactococcus lactis *subsp. lactis; LMHCC_1847 (YP_002350802)_*Listeria monocytogenes *HCC23; AzrA (AB263756)_*Bacillus *sp. B29; AcpD (YP_252301)_*Staphylococcus haemolyticus *JCSC1435; AcpD (YP_039668)_*Staphylococcus aureus *subsp. aureus MRSA252; AcpD (YP_149218)_*Geobacillus kaustophilus *HTA426; YvaB (NP_391234)_*Bacillus subtilis *subsp. subtilis strain 168; YvaB (YP_001422634)_*Bacillus amyloliquefaciens *FZB42; AzoR (AAG04174)_*Pseudomonas aeruginosa *PAO1; AZOR (Q8X9S9)_*Escherichia coli *O157:H7; AzoR (1V4B_A)_*Escherichia coli*; Azo1 (AY545994)_*Staphylococcus aureus *ATCC 25923; Azo1 (Q4L3N6)_*Staphylococcus haemolyticus *JCSC1435; SERP0206 (YP_187802)_*Staphylococcus epidermidis *RP62A; AZR (ACF54629)_*Staphylococcus cohnii *AZR; L8106_10307 (ZP_01622489)_*Lyngbya *sp. PCC 8106; GYMC10DRAFT_0416 (ZP_03036583)_*Geobacillus *sp. Y412MC10;Azr (AB032601)_*Bacillus *sp. OY1-2; YB0110 (AY150311)_*Rhodobacter sphaeroides*; AZR (AB071368)_*Bacillus subtilis *ISW1214; AZR (AB071367)_*Geobacillus stearothermophilus *IFO13737; AzoB (GQ247527)_*Pigmentiphaga Kullae *K24; AzoA k24 (AY165002)_*Pigmentiphaga Kullae *K24; EU307209 (ACA34616)_*Erwinia chrysanthemi*; RSMK02502 (YP_002254667)_*Ralstonia solanacearum *MolK2; H16_A2352 (YP_726815)_*Ralstonia eutropha *H16; RALTA_A1896 (YP_002005902)_*Cupriavidus taiwanensis *strain LMG 19424; AzoB (AF466104)_*Xenophilus azovorans *KF46F.

Figure 4 shows the result of phylogenetic analysis for azoreductases with the NAD(P)H binding domain information. The tree shows three distinct groups for the 30 azoreductases or hypothetic azoreductases. The polymeric flavin-dependent enzymes were further divided into two groups, NADPH-preferred and NADH-preferred azoreductases, respectively. Collectively, the first is the polymeric flavin-dependent NADH-preferred azoreductase group, which contains AzoA from *E. faecalis *[9], AzoR from *E. coli *[14], etc. The second is the polymeric flavin-dependent NADPH-preferred azoreductase group. Azo1 from *S. aureus *[12] and Azr from *Bacillus *sp. OY1-2[7], etc. belong to this group. The third group contains several monomeric flavin-free NADPH-preferred azoreductases, such as AzoA from strain K24 [17].

*P. kullae *K24 AzoB belongs to the flavin-free azoreductase group that displays over 39% sequence identity to each in the third group, with the exception of AzoB from *X. azovorans *KF46F, which shows very low sequence identity with other azoreductases (< 14%). The PD values within each group were less than 0.788. While the overall degree of amino acid sequence identity between groups is no more than 18%, members in each group show over 23% sequence identity to one another. A key fingerprint motif for NAD(P)H binding was identified at the N-terminus of the deduced amino acid sequences of the flavin-free azoreductase group. Sequence alignment of flavin-free azoreductases revealed three conserved Glys (GXXGXXG) and an Arg that are known to govern dinucleotide recognition [19,20]. In flavin-dependent NADPH-azoreductase group, the majority of enzymes show the glycine-rich pyrophosphate-binding motifs GXGXXG or GXXGXXG (data not shown). However, any type of glycine-rich pyrophosphate binding motifs was not conserved in the protein sequences of flavin-dependent NADH-azoreductase group.

**Figure 5:**
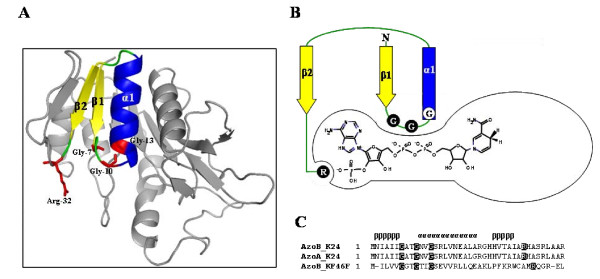
**Structural representation of *P. kullae *K24 AzoB for dinucleotide binding**. A, shows cartoon structure of AzoB, in which the key residues for NADPH binding are colored. B, in the AzoB-NADPH complex, there is a wide exposed cleft adjacent to the nicotinamide moiety. C, the alignment of the glycine-rich pyrophosphate-binding sequences from the enzymes studied. The secondary structure assignment is based on the AzoB. The residues related to NADPH binding are boxed.

3D structural model of AzoB from strain K24 was constructed using the crystal structure (3DHN) of the putative epimerase from *Bacteroides thetaiotaomicron *as a template. RMSD (Cα) of the AzoB model (202 amino acids) superimposed on the 3DHN (216 amino acids) is 0.405 Å, in which 197 amino acid residues were aligned with 42% sequence identity. In AzoB, the pyrophosphate-binding loop of three conserved glycine residues arranged as G_7_xxG_10_xxG_13 _connects the C-terminus of β1 with the N-terminus of α1 (Figure 5A and 5B). Especially, Arg is in a favorable position for interaction with the monophosphate at the 02' position of adenine ribose in NADPH (Figure 5B) [19].

In SAS analysis of AzoB, three bacterial unknown functional proteins, 3DHN from *B. thetaiotaomicron*, 3EW7 from *Listeria monocytogenes*, and 3H2S from *Lactobacillus casei *showed the highest Smith-Waterman scores with sequence identity of 43%, 35.3%, and 32%, respectively. Despite a relatively low sequence identity (28%) with amino acid overlap of 205, a human biliverdin IXβ reductase (BVR-B, 1HDO), which is an early fetal bilirubin IXβ producing enzyme, shows appreciable overall alignment of secondary structure with AzoB. RMSD of 1HDO (205 amino acids) superimposed on AzoB (202 amino acids) is 1.41 Å on 165 residues aligned with 17% sequence identity [21].

## Discussion

In our effort to study the three dimensional structure of flavin-independent azoreductase and mechanism of azo dye reduction by the enzyme, we characterized a novel azoreductase gene encoding a flavin-free azoreductase from *P. kullae *K24. Cell-extract from the recombinant *P. kullae *K24 AzoB revealed an azoreductase activity of 6-fold higher than that of cell-extract from the recombinant AzoA. The specific activity of the purified AzoB is about 3-fold higher than that of AzoA. These data demonstrated that AzoB is facile to be expressed and the enzyme is very efficient in reducing Orange I compared to AzoA from *P. kullae *K24 [17]. Among the tested azo dyes, AzoB is only able to reduce Orange I and not the monoazo dye Magneson II. On the other hand, AzoA has minor activity against Magneson II (13% of that Orange I).

AzoB requires 2 mol of NADPH as four electron donor for the complete reductive cleavage of Orange I to sulfanilic acid and 1-amino-4-naphthaol. Attempts to orient NADPH in AzoB were guided by two criteria. First, the three conserved glycine residues Gly-7, 10, and 13 interact with the pyrophosphate of NADPH. Second, Arg-32 interacts with the 2'-phosphate, providing an anchor for the 2'-phophate-AMP half of NADPH, defining the coenzyme specificity. The presence of at least one Arg side chain in the vicinity of the 2'-phosphate of NADP is a common feature of NADP dinucleotide binding fold complex [20]. In contrast, it is known that NAD- and FAD-binding Rossmann fold proteins have an acidic amino acid residue of Asp or Glu in the position where the functional group carboxylate hydrogen bonds to the 2' hydroxyl of the adenine ribose [19]. The information addresses the limitation on availability of NADH in AzoB, although the enzyme utilizes both NADH and NADPH. As shown in Figure 5, when bound in the active site of AzoB, the nicotinamide ring of NADPH is located in a cleft large enough to accept the substrate Orange I. The suggested NADPH binding mode and putative substrate binding site of the AzoB:NADPH binary complex support the idea that AzoB joins two substrates, Orange I and NADPH, together, in the active site, in which the reaction center of Orange I lies near to the reactive C4 of the NADPH, an appropriate position for direct hydride transfer rather than a proton-relay catalytic reaction. Bibliomic data strongly supports the proposed catalysis model [21]. A human monomeric NADPH-dependent biliverdin IXβ reductase (BVR-B), with its overall structure that is closely related to AzoB, has adopted the same reaction mechanism, in which the substrates, NADPH and biliverdin share a common binding site for direct hydride transfer from the C4 of the dinucleotide [21].

There is a clear correlation between structure, cofactor requirement, and substrate specificity in azoreductases. Among the tested azo dyes, AzoB is only able to degrade Orange I, and other monomeric flavin-free azoreductases also show very narrow substrate specificity [17,22]. On the other hand, polymeric flavin-dependent azoreductase families can catalyze substrates which vary in both chemical nature and size [5,7,9,12,13,23,24]. Structural characteristics in the active sites appear to dictate the substrate diversity of the enzymes. For example, the dimeric AzoA from *E. faecalis *has two separate active sites located at the interfaces between the two monomers, and FMN lies inside each active site, in which *si *face of the isoalloxazine ring provides room for both NAD(P)H and substrate binding for the sequential transfer of four electrons from NAD(P)H to the substrate via FMN [15,25]. The enzyme is not only able to decolorize Methyl Red, but is also able to convert sulfonated azo dyes Orange II, Amaranth, Ponceau BS, and Ponceau S. On the other hand, AzoB has a relatively small substrate binding site, which simultaneously accepts both the nicotinamide ring of NADPH and the substrate in the catalytic cycle. Moreover, the demands for successful direct hydride transfer between the two substrates require more sophisticated binding mode. Presumably because of these constraints, the number of substrates catalyzed by the monomeric azoreductases is limited. A survey of the efficiency of various azo dyes as substrate for an Orange II flavin free azoreductase from *X. azovorans *KF46F revealed that a hydroxygroup in the 2-position of the naphthol ring of azo dyes is required and it is only able to reduce Orange II and its analogues [16,22]. AzoA from *P. kullae *K24 converted only azo dyes that carried a hydroxyl group in the 4-position of the naphthol ring relative to the azo group [18].

Bacterial oxygen-insensitive azoreductases can be classified into at least three distinct non-homologous groups, based on structure, flavin dependency, and dinucleotide preference. Phylogenetic analysis also mirrors well the grouping scheme at the molecular level. The cofactor preference further divides the polymeric azoreductases into two different groups, and the third group is strikingly different from the two polymeric azoreductase groups in respect of both its structural and biochemical requirements for catalytic process. Based on its biochemical and phylogenetic relationship, the enzymes of monomeric azoreductase group seem to have a different origin but have developed towards the same chemical function of azoreduction, suggesting convergent evolution. Nevertheless, significant difference in biochemistry and structure indicates that the monomeric azoreductase group has adopted different catalytic strategies from that of the polymeric azoreductase groups.

## Conclusion

AzoB from strain K24 is a member of the monomeric flavin-free NADPH-preferred azoreductase group. Biochemical analysis and homology modeling studies of AzoB demonstrated how NADPH is recognized and oriented in the active site. Our data indicated a narrow substrate specificity of the enzyme. Phylogenetic analysis revealed that the oxygen-insensitive azoreductases can be divided into three distinct groups. Further investigations on the protein crystallization and mutant experiments to obtain decisive proof of the proposed biochemistry of AzoB from strain K24 are warranted.

## Methods

### Bacterial strains, plasmids, and growth conditions

*P. kullae *K24 ATCC ***BAA-795 ***was grown in Trypticase Soy Broth (TSB) or on TSB agar plates at 30°C for 48 h and used for inoculum and genomic DNA preparation. *E. coli *TOP10F' (Invitrogen), NovaBlue (DE3) (Novagen), and BL21-Gold(DE3)pLysS (Stratagene) were used for recombinant DNA studies. *E. coli *strains were cultured at 37°C in Luria-Bertani (LB) medium with appropriate antibiotics (50 μg/ml). The plasmids pCR2.1-TOPO (Invitrogen) and pET-11a (Stratagene) were used for cloning and expression, respectively.

### Cloning of *P. kullae *K24* azoB *gene and expression of AzoBin *E. coli*

Genomic DNA of *P. kullae *K24 was isolated based on a similar method described by Wang et al. [26]. Plasmids from *E. coli *Top10F' and NovaBlue (DE3) were isolated using a Qiaprep Spin Miniprep kit (Qiagen). A DNA fragment containing the putative *P. kullae *K24 azoreductase gene was obtained by PCR with the genomic DNA of *P. kullae *K24 as template. The forward primer included an *Nde*I site before the start codon: 5'-gattcatatgaatatcgccatcatcggc-3' (K24NEW-forward). The reverse primer included a *Bam*HI site down stream of *azoB*: 5'-cgggatccgcgctgatggccaagaggcc-3' (K24NEW- reverse). The PCR primers were designed based on GenBank accession number AY165002. PCR was performed in a Mastercycler gradient (Eppendorf) and amplification conditions were one cycle of 95°C for 3 min, 30 cycles with each cycle including 30 s of melting at 95°C, 40 s of annealing at 50°C, and 60 s of extension at 72°C, and one final extension cycle at 72°C for 15 min. The amplicon was examined by 1.5% agarose gel electrophoresis.

The amplicon recovered from the gel was directly cloned into pCR2.1-TOPO vector and sequenced. For over-expression of native AzoB in *E. coli*, the amplicon was cleaved with *NdeI *and *Bam*HI (New England BioLabs). The digested DNA was purified from agarose gel and ligated into pET-11a with a rapid DNA ligation kit (Roche). The resulting plasmid pAZOB was transformed to *E. coli *NovaBlue (DE3). The plasmids (pAZOB) were subsequently isolated and introduced into *E. coli *BL21-Gold(DE3)pLysS. DNA sequence analysis, translation, and alignment with related genes and proteins were carried out using the Lasergene program (Version 8, DNASTAR). The GenBank program BLAST was utilized to find similar genes or proteins.

### Enzyme assay

Azoreductase activity was measured spectrophotometrically by monitoring the reduction of Orange I at 482 nm (ϵ_482 _= 22.3 mM/cm) at room temperature. The reaction mixture was in 1.0 ml 50 mM potassium phosphate buffer (pH 6.8), containing 200 μM NADH or NADPH, 25 μM Orange I, and the enzyme. One unit of activity was defined as the amount of enzyme needed for the reduction of 1 μmol Orange I per min. Proteins were quantified using the bicinchoninic acid assay (Pierce) with bovine serum albumin (BSA) as the standard. Some azo dyes including Methyl Red, Amaranth, Ponceau BS, Ponceau S, Orange II, Orange G, 4-(4-Nitrophenylazo)-1-naphhol (Magneson II), 1-(4-Nitrophenylazo)-2-naphthol, and 4-(4-Nitrophenylazo)-resorcinol also served as substrates.

### Purification of azoreductase of *P. kullae *K24from the recombinant *E. coli*

Induction of target protein was performed using a similar procedure as described previously [9]. Protein purification was performed at 4°C using an AKTApurifier 10 system with UNICORN 4.10 software (GE Healthcare). Three liters of the recombinant strain culture was centrifuged at 3,200 × *g*, 10 min. The collected cells were disrupted by freezing and thawing followed by 5 min sonication at 4°C with a vibracell VCX 400 model sonifier. Cell debris was removed by centrifugation at 12,000 × *g *for 10 min. Azide was added to the supernatant (crude enzyme) to a final concentration of 0.02% (w/v). Ammonium sulfate was added to the crude enzyme to a final concentration of 0.5 M. The mixture was centrifuged and filtered using a 0.2 μm syringe filter. The enzyme was applied to a HiPrep 16/10 phenyl FF column. The column was eluted as follows: (1) 40 ml of 0.5 M (NH_4_)_2_SO_4_, (2) 40 ml of 0.05 M (NH_4_)_2_SO_4_, and (3), 40 ml of water. All of the active fractions were collected, concentrated and diluted by 20 mM phosphate buffer (pH 6.8). The fraction was applied to a SP XL column. The column was eluted as follows: (1) 40 ml of 20 mM phosphate buffer, (2) 40 ml of linear gradient to 0.2 M NaCl, (3) 40 ml of 1 M NaCl. All of the active fractions were collected.

### SDS-PAGE analysis

SDS-PAGE was carried out in Laemmli's buffer [27] with 12.5% polyacrylamide. Perfect protein markers (Novagen) were used. Electrophoresis was performed in a Hoefer SE 260 Mighty Small II Mini Vertical Unit (GE Healthcare). Gels were stained for proteins with Coomassie brilliant blue R-250 (Bio-Rad).

### Apparent kinetic constants of azoreductase from the recombinant *E. coli *contained *azoB *gene from *P. kullae *K24

Initial velocities of the enzymatic reaction were performed by varying concentrations of one substrate, Orange I (from 1.5 to 15 μM) or NADH/NADPH (from 0.07 to 0.7 mM), while the concentration of the other substrate was kept constant (NADH/NADPH: 1 mM or Orange I: 30 μM). Apparent *K*_m _and *V*_max _values were obtained from Lineweaver-Burk plots.

As for the optimum pH, the enzyme activities were carried out in 50 mM Sorensen's phosphate buffer with pH values ranging from pH 4.6 to pH 7.6. To determine the optimum temperature of the enzyme, optical density (OD _482 nm_) was detected before NADH/NADPH was added to the mixture. When NADH/NADPH was added (200 μM), the mixture was incubated at different temperature (25°C, 30°C, 37°C, 40°C, 45°C, 50°C, 55°C, and 60°C) for 5 min, and then the optical density (OD _482 nm_) was detected immediately.

### *In silico *analysis

ClustalX [28] was used to obtain multiple sequence alignments, which the grouping and comparison of azoreductases were conducted in ClassRHO [29]. Phylogenetic analyses were conducted in MEGA4 [30]. The homology model of AzoB was generated using the Swiss-Model server [31] with the 3DHN as a template. Basic structure validation was checked using PROCHECK (European Bioinformatics Institute, Cambridge, U.K). The pocket volumes of AzoB were measured using both CASTp [32] and Pocketfinder http://bmbpcu36.leeds.ac.uk/pocketfinder. SAS (Sequence Annotated by Structure, http://www.ebi.ac.uk/thornton-srv/databases/sas/) was used to apply structural information to AzoB amino acid sequence. RMSD (Root-Mean-Square-Deviation) was calculated by SuperPose (Ver. 1.0) [33]. PyMOL (0.99RC6, http://www.pymol.org) was used to visualize the 3D structures.

### Nucleotide sequence accession number

The nucleotide sequence of *azoB *of *P. kullae *K24 has been assigned accession number **GQ247527 **in GenBank database.

## Authors' contributions

All authors participated in the design of the study and writing the paper. HC and CEC directed the whole research and critically revised the paper. All authors read and approved the paper.
